# Legendary fermented herbs: an ethnobotanical study of the traditional fermentation starter of the Chuanqing people in Northwestern Guizhou, China

**DOI:** 10.1186/s13002-024-00708-6

**Published:** 2024-09-10

**Authors:** Jiawen Zhao, Qinghe Wang, Zixuan Ren, Changqin Yang, Shiyu Guan, Xiaoyan Wang, Yan Huang, Ruyu Yao, Hongxiang Yin

**Affiliations:** 1 School of Ethnic Medicine, Chengdu University of Traditional Chinese Medicine, Chengdu, 611137 China; 2 School of Pharmacy, Chengdu University of Traditional Chinese Medicine, Chengdu, 611137 China; 3 Institute of Botany, Jiangsu Province and Chinese Academy of Sciences, Jiangsu, 210014 China; 4 Kunming Institute of Botany, Chinese Academy of Sciences, Kunming, 650201 China

**Keywords:** *Jiuqu*, Microbial community, Ethnobotany, Traditional knowledge, Cultural protection

## Abstract

**Background:**

Plants that contain brewing microorganisms are used in traditional fermentation starters, which are an essential part of local diet, nutrition, life, and health. Regionally, the plant species used and the microorganisms included in traditional fermentation starters are diverse, endowing local fermented drinks with different flavors and health benefits. However, related traditional knowledge has been scarcely documented or revealed.

**Methods:**

An ethnobotanical survey was conducted in five towns of Nayong County in northwestern Guizhou, China. Snowball sampling, semi-structured interviews, free lists, and participatory observation were used to collect information on *Jiuqu* Plants (JPs) and *jiuqu*-making techniques. The PacBio platform was used to study the microbial community structure and diversity in the Chuanqing people’s *jiuqu*.

**Results:**

In total, 225 informants were interviewed, including 116 who provided plants and technological processes for making Chinese *baijiu jiuqu* (CBJ) and 139 who provided information about making fermented glutinous rice *jiuqu* (FGRJ). This study found that older people have more abundant knowledge about CBJ plants. Poaceae was found to be the dominant family used in making CBJ and FGRJ (7 species each). Compared to individual plant parts, the whole plant is most commonly used in two kinds of *jiuqu* (19.5% in CBJ and 22.6% in FGRJ). The Chuanqing people’s *jiuqu* is used to treat dietary stagnation and indigestion. The highest relative frequency of citation of the CBJ plant was *Ficus tikoua* Bureau, and the counterpart of the FGRJ plant was *Buddleja macrostachya* Benth. The dominant bacterial species in *jiuqu* were *Gluconobacter japonicus* (YQ1, YQ4) and *Pediococcus pentosaceus* (YQ2, YQ3), and the dominant fungal species was *Rhizopus oryzae.*

**Conclusion:**

For the first time, this study documents the unique traditional *jiuqu* knowledge and reveals the microbial mystery behind the FGRJ of the Chuanqing people.

Therefore, this study encourages the use of online social media platforms in order to spread *Jiuqu* culture, the use of the new media wave in order to create multimedia databases, and also suggests that local communities should develop preservation intervention programs, in addition to nurturing the inheritors in order to prevent the disappearance of traditional *Jiuqu* knowledge. This research contributes to the conservation and demystification of the traditional *jiuqu* knowledge of the Chuanqing people and lays the foundation for further research on its microbiology, nutrition, and metabolomics.

## Introduction

Fermentation is everywhere, all the time 25. As one of the oldest and most economical processing methods, fermenting food has been employed for millennia to extend shelf life, improve flavor and taste, and increase food products’ nutritional and medicinal value [4, 47]. Fermentation in particular draws attention to artisanal food production, flavor and identity, as well as to the practice of traditional ecological knowledge [10]. Fermented products are an essential part of the global human diet, nutrition, life, and health [9, 16], [28, 30, 46, 54]. Moreover, fermented products hold great promise for meeting the world’s growing food demand, income increases, and employment opportunities [1]. *Baijiu* (a distilled spirit originating from China) is one of the most important fermented products in China, which not only plays an important role in traditional Chinese culture, but also contributes significantly to healthcare and economic development [2, 6, 34].

*Jiuqu* (fermented cereal and plants that contain brewing microorganisms) is used as a starter in Chinese brewing, marking point of departure between Chinese and Western brewing cultures, as it is not employed in Western brewing [11, 12, 21]. The fabrication procedures of *jiuqu* originated in China before the Shang Dynasty (1600 BCE) and were then transmitted to South Korea, Japan, and Vietnam [72]. In the brewing process, *jiuqu* mainly provides enzymes for the fermentation of alcoholic drinks, which undergo a simultaneous saccharification and fermentation process, as well as the production of flavor and precursor compounds [21]. Alcoholic beverages are closely linked to herbal traditions [39, 66]. The approach of adding herbs during the fermentation process was first recorded in *Nanfang Caomu Zhuang* (A book on the flora of Lingnan, the earliest dated botanical journal of Lingnan) (Ji, A.D.304). Studies have suggested that adding herbs provides microorganisms, protein, flavor components, and bacteriostatic ingredients from plants, which can inhibit harmful microorganisms and increase the taste, nutrition, and effects of alcoholic drinks [40, 55, 60].

To date, few systematic studies have been conducted regarding the traditional knowledge and skills about fermented *jiuqu* from ethnic minorities, such as those of the Chinese Dong, Shui, and Miao ethnic groups, and the use of *jiuqu* by other ethnic minorities has been largely ignored [18, 20], [51, 70]. The Chuanqing people are a native ethnic group in China that mainly live in Guizhou Province which is endowed with rich medicinal plant resources and unique history and culture [36, 38, 58]. They worshipped the *Mandrillus sphinx* and believed in the Five Apparent Deities. There are various theories about the origin of the Chuanqing people, one of which is that they were natives of Guizhou during the Ming Dynasty. According to another theory, they are actually the Han nationality immigrants from the early part of history [61]. There are four main types of livelihoods for them: job-oriented, farming-oriented, business-oriented and craft-oriented [62]. The Chuanqing people’s *jiuqu* not only constitutes a vital component of their culture but also plays an essential role in their households’ livelihoods and health care. Compared to pure-bred fermentation technology, it has unique materials, high saccharification, high yield, and a unique aroma. However, facing an aggressive wave of globalization, the Chuanqing people’s knowledge of traditional fermentation has been marginalized due to unprecedented challenges. Thus, the social traditions, legend, folklore, significance, and memories of the Chuanqing people’s knowledge of traditional fermentation could be lost, to the detriment of human cultural heritage. Therefore, recording and protecting regional traditional fermentation knowledge is imperative.

This study aims to document and demystify the ethnobotanical knowledge of traditional *jiuqu* and its traditional processing by the Chuanqing people. Additionally, the effect of *jiuqu* on family livelihood, health care, and cultural customs are explored. This study contributes to the conservation of the traditional fermentation knowledge of the Chuanqing people, provides a new lens for the Chuanqing people’s economic development, and lays the foundation for further research on the microbiology, nutrition, and metabolomics of the Chuanqing people’s *jiuqu*.

## Materials and methods

### Survey area

**Fig. 1:**
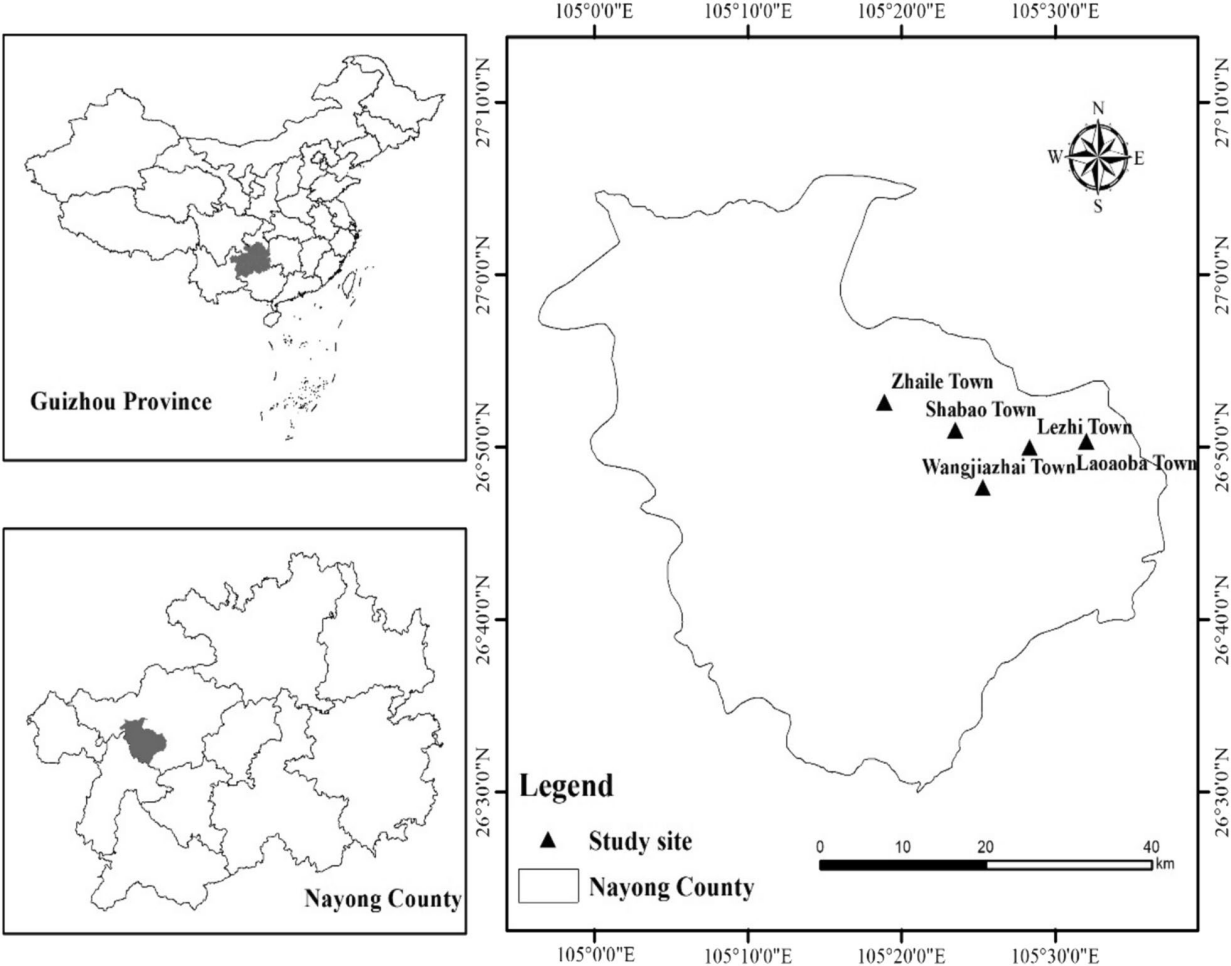
Location of the study area, Nayong County, Guizhou Province, China (ArcGIS 10.4)

The study area was Nayong County, which is located in the northwest of Guizhou, China (26°39′-26°46′ N, 105°17′-105°31′ E) (Fig. 1). Nayong County is the core distribution area of the Chuanqing people, with a population of nearly 300,000. The traditional customs and habits of the county’s Chuanqing people are very well preserved, and the ethnic characteristics of the Chuanqing people’s traditional culture and medical knowledge are distinct and representative. Nayong County is the transition zone between the Yunnan-Guizhou Plateau and the central mountain plain in the middle of Guizhou, and is on the southern slope of Wumeng Mountain [73]. According to data recorded by the Bijie Meteorological Bureau in China, the county comprises 22,500 km^2^ and 29 small towns. According to the National Meteorological Centre (http://www.nmc.cn/), the survey area has a subtropical monsoon climate with a mild climate, no severe cold in winter and no heat in summer, with average summer temperatures ranging from 23 to 26 °C, and average winter temperatures ranging from 7 to 10 °C. The average annual temperature is 13.6 °C, and rainfall is 1250 mm. The elevation ranges from 1074 to 2447 m. Regarding topography, Nayong County is located in the karst zone. According to official data, the vegetation in the area is mainly evergreen deciduous mixed forest [32]. A total of 1857 plant species from 277 families and 772 genera, along with 174 species of wild vertebrates from 56 families and 26 orders, have been recorded in Nayong County [15].

The survey for the current study was conducted from 2017 to 2020. A total of 16 villages in five townships were investigated, comprising the main settlements of the Chuanqing people.

### Ethnobotanical survey and data analysis

During the Dragon Boat Festival survey in June 2017, the researchers found that some herbs used for making the Chuanqing people’s *jiuqu* were sold and that alcoholic drinks played an essential role in the lives of the Chuanqing people—for example, the roots of *Chloranthus henryi* Hemsl were soaked in alcohol to treat internal lesions caused by overexertion [58]. Moreover, the local people were found to prefer self-brewed drinks, mainly those including Chinese *baijiu* and fermented glutinous rice. This *jiuqu* is different from that used in the modern liquor industry in China, so it can be viewed as a typical case of the ethnobotanical characteristics of the Chuanqing people [58].

In our study, the gender, age, occupation and education level of the informants were recorded using snowball sampling method, as well as the place name, scientific name, family, life form, usage scenario and voucher number of the plants. Subsequently, the production process of CBJ and FGRJ was photographed and documented. These informants had rich botanical knowledge of making *jiuqu* and were familiar with the production process. Then, information on the *jiuqu* plants (JPs) and the technological process were collected using semi-structured interviews, free lists, and participatory observation. The interview questions included the following: (1) Which plants and parts are needed to make *jiuqu*? (2) Where do you collect these plants? Can you describe the characteristics of the plant? (3) How do you know that these plants are used to make *jiuqu*? (4) How are these plants incorporated into the production of *jiuqu*? (5) What is the specific process for making *jiuqu*? We adjusted the questions based on the interviewees and the situation to obtain more information. The interviews followed the Code of Ethics of the International Society of Ethnobiology [22]. Participatory observation was used to record the process of producing *jiuqu*.

After sorting the data, with the help of local herbalists and medicinal farmers, specimens were collected in the field or purchased from herb sellers. The specimens were identified by Dr. Hongxiang Yin of Chengdu University of Traditional Chinese Medicine and Associate Professor Yuxiang Shen of Anshun College using *Flora of China* and *Flora of Guizhou*. The voucher specimens were then stored in the Herbarium of Chengdu University of Traditional Chinese Medicine (CDCM).

The relationship between gender, education level, occupation, and number of JPs was analyzed using Microsoft Excel 2016. The relationship between age and the number of JPs was analyzed using simple linear regression. Quantitative data analysis was conducted using the relative frequency of citation (RFC) 52. The RFC was used to assess the value and importance of a species in the study area. The formula was RFC = FC/N, with FC representing the number of respondents who mentioned a specific species used to make *jiuqu* and N referring to the number of respondents who participated in the survey. Theoretically, the RFC value ranged from 0 to 1. An index of 0 meant that no one believed that a particular species could be used to make *jiuqu*; an index of 1 meant that all respondents mentioned that a particular species could be used to make *jiuqu*. The higher the index value, the more critical the species in the area.

### Microbial community composition analysis

Compared with Chinese *baijiu jiuqu* (CBJ), fermented glutinous rice *jiuqu* (FGRJ) is more widely used in Chuanqing people’s family livelihood and culture; thus, we chose to explore the microbial community composition of the latter. Based on the data collected in the survey, the fruits of *Eleusine coracana* (L.) Gaertn. and *Avena sativa* L. were used by the researchers as substances to make *jiuqu* (labeled YQ2 and YQ3, respectively). The other two samples were bought from local *jiuqu* merchants (labeled YQ1 and YQ4, respectively). The complete DNA was extracted using the TGuide S96 magnetic bead method soil genomic DNA extraction kit (Tiangen Biochemical Technology (Beijing) Co., Ltd., model: DP812). A microplate reader (Synergy HTX, Gene Company Limited) was used to detect and amplify the concentration of nucleic acids.

The concentration of PCR-amplified products was subjected to electrophoresis in a 1.8% agarose gel (Beijing Bomei Fuxin Technology Co., Ltd.). The bacterial 16S rRNA was amplified using universal primers 27F (5’-AGAGTTTGATCMTGGCTCAG-3′) and 1492R (5’-TACGGYTACCTTGTTACGACTT-3′). The fungal ITS1 was amplified using universal primers F (5’-CTTGGTCATTTAGAGGAAGTAA-3’) and R (5’- GCTGCGTTCTTCATCGATGC-3’). The 16S rRNA full-length reaction procedure was as follows: pre-denaturation at 95 ℃ for 2 min, denaturation at 98 ℃ for 10 s, annealing at 55 ℃ for 30 s, extending to 72 ℃ for 1 min 30 s for a total of 25 cycles, extending to 72 ℃ for 2 min, and storing at 4 ℃. The ITS full-length reaction procedures were as follows: pre-denaturation at 95 ℃ for 5 min, denaturation at 95 ℃ for 30 s, annealing at 55 ℃ for 30 s, and extending to 72 ℃ for 45 s for a total of eight cycles, denaturation at 95 ℃ for 30 s, annealing at 55 ℃ for 30 s, extending to 72 ℃ for 45 s for a total of 24 cycles, extending to 72 ℃ for 5 min, and storing at 4 ℃.

The samples were sequenced by single-molecule real-time sequencing technology based on the PacBio third-generation sequencing platform. Effective sequences were obtained by further splicing, filtering, and removing chimeric sequences. Usearch software was used to perform sequence analysis, and sequences with ≥ 97% similarity were assigned to the same operational taxonomic units (OTUs). QIIME2 software was used to assess the samples’ microbial richness and evenness based on alpha diversity indices (including Chao1 Shannon indices and coverage value). Based on the bacterial and fungal species taxonomic levels, R was used to draw species distribution maps and clustered heatmaps of species abundances.

## Results

### Demographic characteristics of informants

**Fig. 2:**
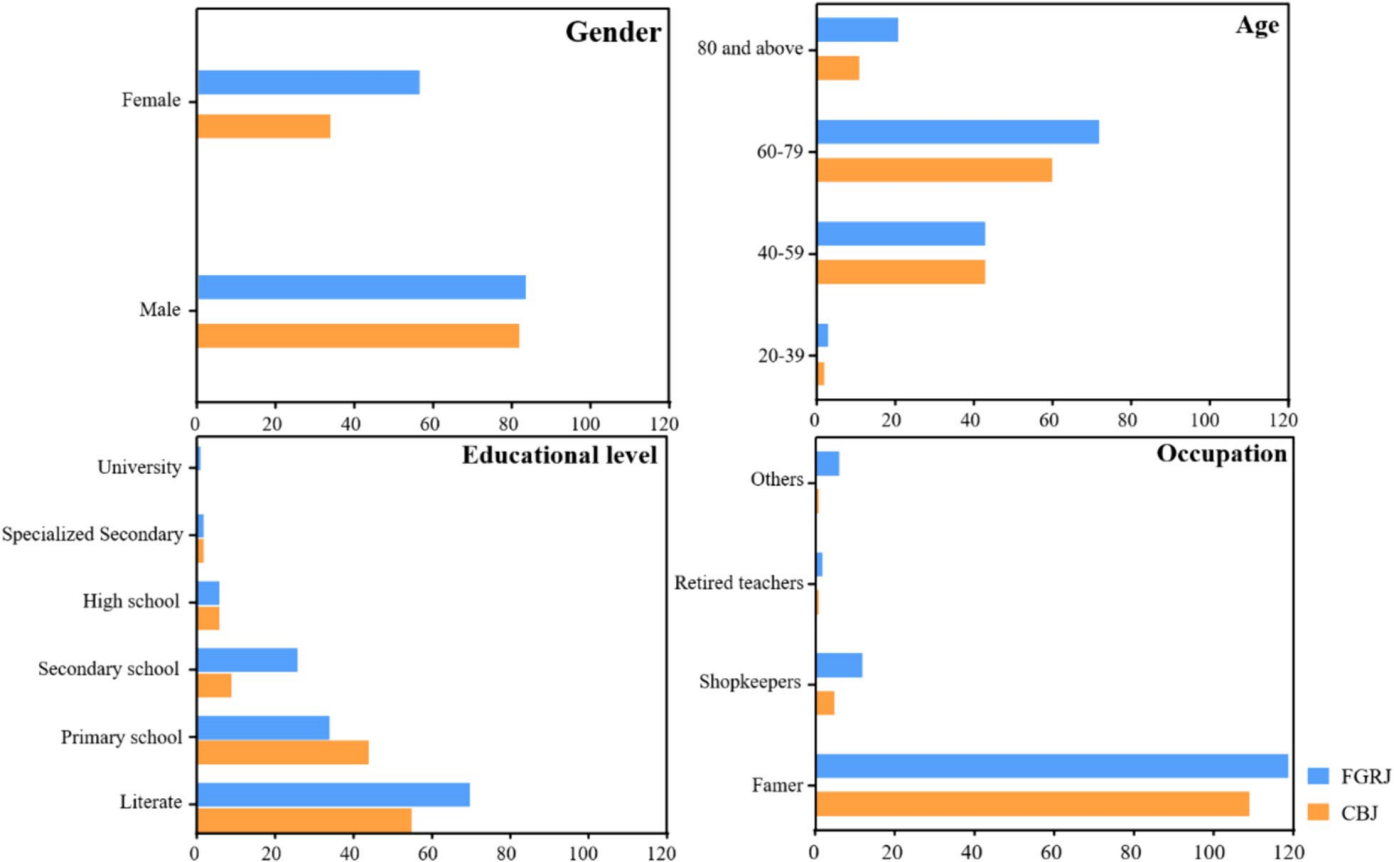
Demographic characteristics of informants

As shown in Table 1 and Fig. 2, a total of 255 informants were interviewed, including 116 who provided information on raw materials and technological processes for making CBJ and 139 who provided information on making FGRJ. A total of 116 informants, including 82 men (70.7%) and 34 women (29.3%), provided plant information for making CBJ. The ages of these informants ranged from 31 to 95 years (61.2%, age ≥ 60). The predominant education level of the informants was literate (47.4%). Of these 116 informants, 109 (93.9%) were farmers. A total of 139 informants, including 83 men (59.7%) and 56 women (40.3%), provided plant information for making FGRJ. The ages of these informants ranged from 20 to 95 years (66.9%, age ≥ 60). The predominant education level of these informants was literate (50.4%). Among these 139 informants, 119 (85.6%) were farmers. The plant knowledge mastered by the men for making *jiuqu* was found to be much higher than that mastered by the women. Moreover, the knowledge of making *jiuqu* was found to be mainly mastered by people aged 60–95 years. Overall, the informants were generally undereducated, and most of them were farmers.

**Table 1:** Informants’ demographic characteristics

Demographic characteristics	CBJ		FGRJ	
	Number	Percentage (%)	Number	Percentage (%)
Gender				
Male	82	70.7	83	59.7
Female	34	29.3	56	40.3
Age				
20–39	2	1.7	3	2.2
40–59	43	37.1	43	30.9
60–79	60	51.7	72	51.8
80 and above	11	9.5	21	15.1
Educational level				
Literate	55	47.4	70	50.4
Primary school	44	37.9	34	24.5
Secondary school	9	7.8	26	18.7
High school	6	5.2	6	4.3
Specialized secondary school	2	1.7	2	1.4
University			1	0.7
Occupation				
Farmer	109	93.9	119	85.6
Shopkeepers	5	4.3	12	8.7
Retired teachers	1	0.9	2	1.4
Others	1	0.9	6	4.3

According to the results of the simple linear regression, the number of JPs used for making CBJ was related to the age of the informants; with an increase in age, the number of medicinal materials mastered gradually increased (R^2^ = 0.1284, *P* < 0.0001). However, no significant correlation was found between the number of JPs for making FGRJ and the age of the informant (R^2^ = 0.008939, *P* > 0.05).

### JPs diversity of the Chuanqing people

**Fig. 3:**
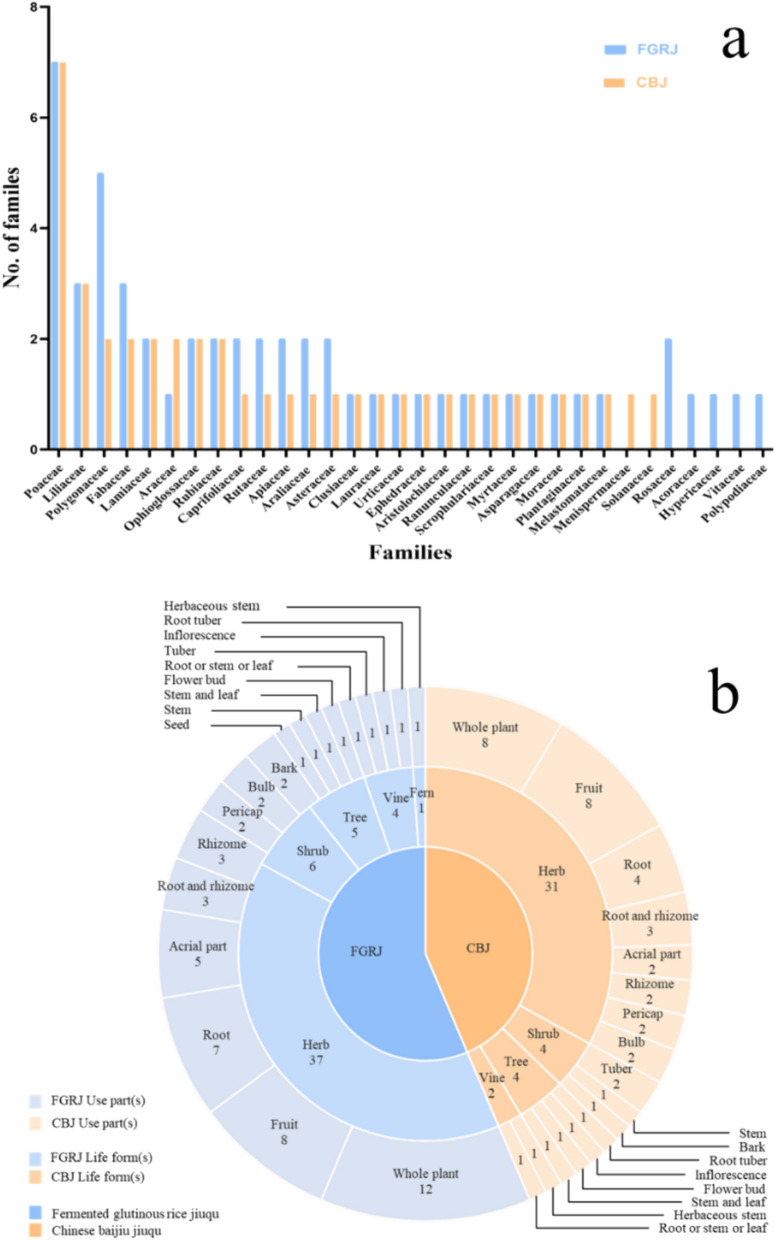
**a** Number of plant families used to make CBJ and FGRJ; **b** number of plant parts and life forms used to make CBJ and FGRJ

Numerous plants were used by the Chuanqing people to make *jiuqu*, with a total of 57 species belonging to 51 genera and 32 families (Table 2). The families, plant parts, and life forms used in the production process differed for CBJ and FGRJ (Fig. 3). Twenty-seven families were used in CBJ. Among them, the most frequently used family was Poaceae (7 species), followed by Liliaceae (3 species), and the remaining families were used only once or twice each. Thirty families were used in FGRJ. Among them, the most frequently used family was Poaceae (7 species), followed by Polygonaceae (5 species), Liliaceae and Fabaceae (3 species each), and the remaining families were used only once or twice each. Seventeen kinds of plant parts were used for CBJ, among which the whole plant was the most frequently used (19.50%), followed by the fruit (19.50%) and the root (9.80%). Eighteen kinds of plant parts were used for FGRJ, among which the whole plant was the most frequently used (22.60%), followed by the fruit (15.10%), the root (9.40%), and the aerial part (5.70%). Regarding habits, CBJ included the highest proportion of herbaceous plants (75.60%), and FGRJ was also same (69.80%).

**Table 2:** List of JPs used by the Chuanqing people in Nayong County, Guizhou, China

Scientific name	Local name	Family	Used part	Life form	RFC-CBJ	RFC-FGRJ	Voucher number
*Achillea wilsoniana* (Heimerl) Hand. -Mazz	Yi Zhi Hao	Asteraceae	Whole plant	Herb	0.04	0.03	SB130
*Aconitum carmichaelii* Debeaux	Hao Zi Tou	Ranunculaceae	Root	Herb	0.06	0.03	SB127
*Acorus macrospadiceus* (Yamamoto) F. N. Wei & Y. K. Li	Di Pi Hui Xiang	Acoraceae	Rhizome	Herb	–	0.01	ZL017
*Arisaema erubescens* (Wall.) Schott	Lao She Bao Gu	Araceae	Tuber	Herb	0.07	0.03	SB126
*Aristolochia cucurbitoides* C. F. Liang	Qing Teng Xiang	Aristolochiaceae	Whole plant	Vine	0.06	0.12	LZ056
*Arrhenatherum elatius* (L.) P.Beauv. ex J.Presl & C.Presl	Xiao Tian Cao	Poaceae	Stem	Herb	0.01	0.06	ZL071
*Asparagus cochinchinensis* (Lour.) Merr	Yi Wo Qu	Asparagaceae	Root	Herb	0.01	0.01	SB160
*Avena sativa* L	Yan Mai	Poaceae	Fruit	Herb	0.13	0.35	LZ027
*Botrychium ternatum* (Thunb.) Sw	Yi Duo Yun	Ophioglossaceae	Whole plant	Herb	0.01	0.01	SB204
*Buddleja macrostachya* Benth	Jiu Yao Hua	Scrophulariaceae	Inflorescence	Shrub	0.3	0.54	LZ008
*Capsicum annuum* L	La Jiao	Solanaceae	Fruit	Herb	0.02	–	JQ051
*Cinnamomum cassia* (L.) J. Presl	Rou Gui	Lauraceae	Bark	Tree	0.1	0.09	LZ057
*Eleusine coracana* (L.) Gaertn	Hong Bai	Poaceae	Fruit	Herb	0.04	0.06	SB171
*Eleutherococcus nodiflorus* (Dunn) S.Y. Hu	Ci Wu Jia	Araliaceae	Stem and leave	Shrub	0.04	0.06	LZ045
*Ephedra equisetina* Bunge	Ma Huang	Ephedraceae	Herbaceous stem	Herb	0.02	0.01	JQ024
*Fagopyrum tataricum* (L.) Gaertn	Ku Qiao	Polygonaceae	Fruit	Herb	0.31	0.14	LZ094
*Reynoutria multiflora* (Thunb.) Moldenke	He Shi Wu	Polygonaceae	Root tuber	Herb	–	0.01	SB299
*Ficus tikoua* Bureau	Di Gua Teng	Moraceae	Whole plant	Vine	0.5	0.4	SB053
*Foeniculum vulgare* Mill	Hui Xiang	Apiaceae	Fruit	Herb	0.02	0.03	SB051
*Gleditsia sinensis* Lam	Zao Jia	Fabaceae	Fruit	Tree	0.01	0.01	LAB042
*Glycine max* subsp. *soja* (Siebold & Zucc.) H.Ohashi	Ye Da Dou	Fabaceae	Seed	Herb	–	0.01	WJZ004
*Glycyrrhiza uralensis* Fisch	Gan Cao	Fabaceae	Root and rhizome	Herb	0.41	0.48	LZ007
*Gonostegia hirta* (Blume ex Hassk.) Miq	Nuo Nuo Xiang	Urticaceae	Root, stem, leave	Herb	0.02	0.03	SB031
*Hedera sinensis* (Tobler) Hand. -Mazz	San Jiao Feng	Araliaceae	Whole plant	Shrub	–	0.04	LZ006
*Hypericum japonicum* Thunb	Xiao Guo Er Huang	Hypericaceae	Whole plant	Herb	0.02	0.04	SB114
*Hypericum monogynum* L	Da Guo Lu Huang	Hypericaceae	Aerial part	Shrub	–	0.01	LZ076
*Imperata cylindrica* (L.) Raeusch	Mao Zhen Cao	Poaceae	Root	Herb	0.41	0.24	LZ040
*Lilium brownii* F. E. Brown ex Miellez	Bai Po He	Liliaceae	Bulb	Herb	0.03	0.01	SB129
*Lilium sulphureum* Baker ex Hook.f	Hong Po He	Liliaceae	Bulb	Herb	0.03	0.01	SB147
*Lonicera confusa* DC	Jin Yin Hua	Caprifoliaceae	Aerial part	Vine	–	0.05	LZ016
*Neolepisorus ovatus f. doryopteris* (Christ)Ching	Da Hei Gen	Polypodiaceae	Whole plant	Fern	–	0.01	WJZ002
*Ophioglossum reticulatum* L	Yi Zhi Jian	Ophioglossaceae	Whole plant	Herb	0.02	0.01	SB292
*Origanum vulgare* L	Xiao Jiu Yao Hua	Lamiaceae	Whole plant	Herb	0.01	0.01	SB230
*Oryza sativa* subsp. *japonica* Kato	Nuo Mi	Poaceae	Fruit	Herb	–	0.03	LZ002
*Osbeckia stellata* Buch. -Ham. ex Ker Gawl	Chao Tian Guan	Melastomataceae	Root	Herb	0.02	0.02	SB159
*Paris polyphylla* Sm	Du Jiao Lian	Melanthiaceae	Rhizome	Herb	0.06	0.03	SB128
*Peucedanum praeruptorum* Dunn	Yi Ma Cai	Apiaceae	Root	Herb	–	0.01	ZL051
*Phellodendron chinense* var. *glabriusculum* C.K.Schneid	Huang Guo Pi	Rutaceae	Bark	Tree	–	0.01	LZ031
*Pinellia ternata* (Thunb.) Makino	Ban Xia	Araceae	Tuber	Herb	0.02	–	JQ023
*Plantago asiatica* L	Che Qian	Plantaginaceae	Whole plant	Herb	0.02	0.06	LZ011
*Pogostemon cablin* (Blanco) Benth	Guang Xiang	Lamiaceae	Aerial part	Herb	0.07	0.03	LAB004
*Persicaria hydropiper* (L.) Delarbre	La La Cao	Polygonaceae	Aerial part	Herb	–	0.01	SB247
*Persicaria orientalis* (L.) Spach	Shui Hong Hua	Polygonaceae	Fruit	Herb	–	0.01	SB052
*Polygonu viviparum* var. *viviparum*	Di Ma Feng	Polygonaceae	Rhizome	Herb	0.02	0.01	ZL029
*Rosa roxburghii f. normalis* Rehder & E.H. Wilson	Ci Li Gen	Rosaceae	Root	Shrub	–	0.01	SB011
*Rubia cordifolia* L	Xiao Xue Teng	Rubiaceae	Root and rhizome	Herb	0.03	0.03	LZ083
*Rubia salicifolia* H.S.Lo	Da Ju Ju Cao	Rubiaceae	Root and rhizome	Herb	0.01	0.02	LZ060
*Senecio scandens* Buch. -Ham. ex D. Don	Jiu Li Guang	Asteraceae	Whole plant	Herb	–	0.04	LZ005
*Spiraea japonica* L. f	Qiang Dao Jiu Gan Zi	Rosaceae	Root	Shrub	–	0.01	ZL016
*Syzygium aromaticum* (L.) Merr. & L.M.Perry	Ding Xiang	Myrtaceae	Flower bud	Tree	0.13	0.07	LZ095
*Tetrastigma obtectum* (Wall. ex M.A. Lawson) Planch. ex Franch	Yan Wu Jia	Vitaceae	Whole plant	Vine	–	0.01	LZ097
*Tinospora sagittata* Gagnep	Shan Ci Gu	Menispermaceae	Root tuber	Herb	0.01	–	JQ037
*Triticum aestivum* L	Mai Fu	Poaceae	Pericarp	Herb	0.02	0.01	SB207
*Triticum aestivum* L	Xiao Mai	Poaceae	Fruit	Herb	0.02	–	JQ052
*Valeriana jatamansi* Jones	Zhi Zhu Xiang	Caprifoliaceae	Aerial part	Herb	0.04	0.14	LZ010
*Zanthoxylum bungeanum* Maxim	Hua Jiao	Rutaceae	Pericarp	Tree	0.11	0.04	LAB016
*Zea mays* L	Bao Gu	Poaceae	Fruit	Herb	0.01	0.01	SB176

RFC-CBJ: Relative of frequency citation of Chinese Baijiu Jiuqu; RFC-FGRJ: relative of frequency citation of fermented glutinous Rice Ji

The China Rare and Endangered Plant Information System (http://www.iplant.cn/rep/) was searched for information on the protection of JPs, and *Ephedra equisetina* Bunge, *Glycyrrhiza uralensis* Fisch., *Paris polyphylla* Sm., and *Lilium sulphureum* Baker ex Hook.f. were all found to belong to China’s national protection level II. The Chuanqing people use rich plants to make *jiuqu*, which reflects their deep understanding of the natural environment and surrounding plants.

### Medicinal uses for the Chuanqing people’s jiuqu

As local traditional medicine, the Chuanqing people’s *jiuqu* is used to treat dietary stagnation and indigestion, as it strengthens the spleen and harmonizes the stomach by eliminating food and resolving stagnation. To use the *jiuqu*, a small amount of water is added to soften the *jiuqu* before the patient takes it. It is usually taken two or three times to relieve symptoms and four or five times to cure them. No contraindications were reported for taking it. Meanwhile, a small number of people also use *jiuqu* to treat dizziness. In addition, *jiuqu* is also suitable for livestock; for example, it is used to treat diarrhea and swollen bladders in pigs.

Some JPs were also used by the Chuanqing people to treat ailments [58] (Table 3). For example, *Aconitum carmichaelii* Debeaux was used to treat noxious sores and restore yang for resuscitation; *Aristolochia cucurbitoides* C. F. Lian g was used to relieve pain; and *Gleditsia sinensis* Lam. was used to treat osteodynia, arthralgia, and so on.

**Table 3:** Medicinal uses of JPs

Scientific name	Medicinal use
*Aconitum carmichaelii* Debeaux	Noxious sores, restoring yang for resuscitation
*Aristolochia cucurbitoides* C. F. Liang	Relieve pain
*Gleditsia sinensis* Lam	Osteodynia, arthralgia
*Paris polyphylla* Sm	Cardiopathy, sores, swelling
*Peucedanum praeruptorum* Dunn	Diaphoresis
*Phellodendron chinense* var. *glabriusculum* C.K.Schneid	Cooling heat and drying dampness
*Polygonu viviparum* var. *viviparum*	Dysentery
*Rubia cordifolia* L	Relax the veins, stimulate blood circulation
*Spiraea japonica* L. f	Skin disease
*Tinospora sagittata* Gagnep	Neck pain, laryngitis, dysentery, abdominal pain

### RFC analysis of the Chuanqing people’s JPs

**Fig. 4:**
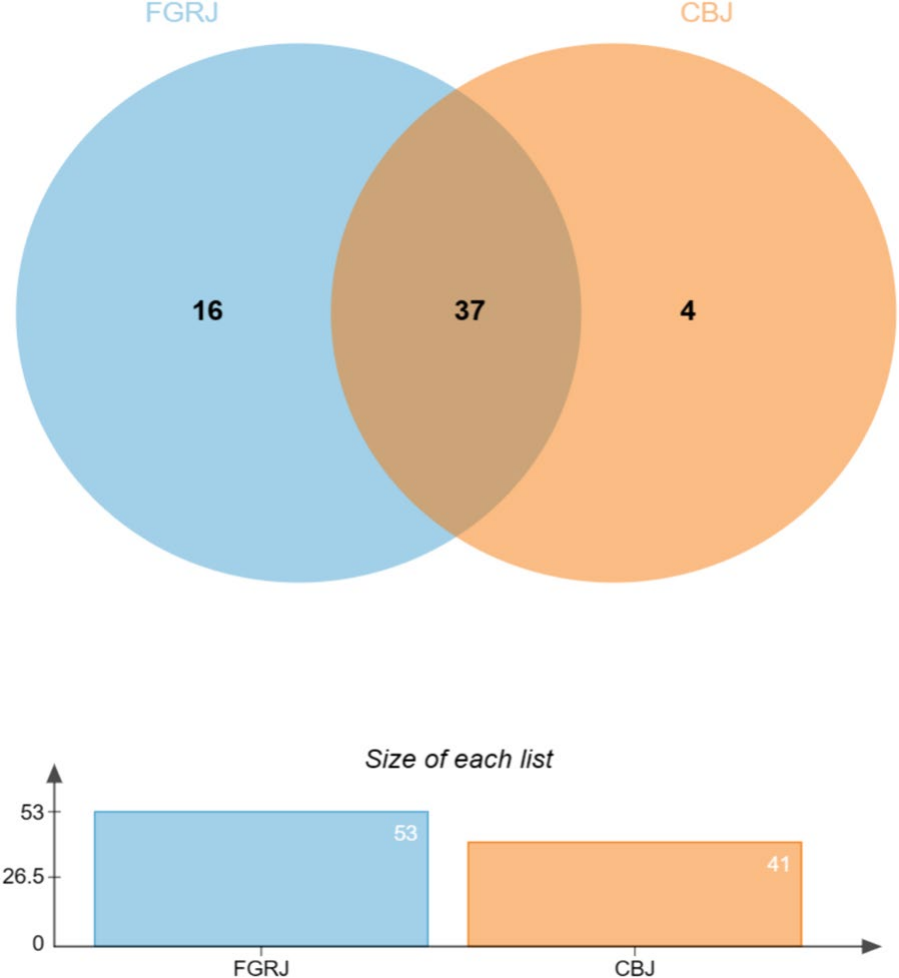
The Venn diagram of numbers of JPs used by Chuanqing people

A total of 41 species plants were used to make CBJ, and 53 species were used to make FGRJ, with a total of 37 plants used in both species of *jiuqu* (Fig. 4). The results showed that the RFC values of the 41 CBJ plants ranged from 0.01 to 0.50, and nine had an RFC over 0.1: *Cinnamomum cassia* Presl (0.10), *Zanthoxylum bungeanum* Maxim. (0.11), *Syzygium aromaticum* (L.) Merr. & L.M.Perry (0.13), *Avena sativa* L. (0.13), *Buddleja macrostachya* Wall. ex Benth. (0.30), *Fagopyrum tataricum* (L.) Gaertn. (0.31), *Glycyrrhiza uralensis* Fisch. (0.41), *Imperata cylindrica* (L.) Raeusch. (0.41), and *Ficus tikoua* Bureau (0.50). The RFC values for the 53 FGRJ plants ranged from 0.01 to 0.54, and eight had an RFC over 0.1: *Aristolochia cucurbitoides* C. F. Liang (0.12), *Valeriana jatamansi* Jones (0.14), *Fagopyrum tataricum* (L.) Gaertn. (0.14), *I. cylindrica* (L.) Raeusch. (0.24), *Avena sativa* L. (0.35), *Ficus tikoua* Bureau (0.40), *Glycyrrhiza uralensis* Fisch. (0.48), and *B. macrostachya* Wall. ex Benth. (0.50). *Fagopyrum tataricum* (L.) Gaertn., *I. cylindrica* (L.) Raeusch., *Avena sativa* L., *Ficus tikoua* Bureau, *Glycyrrhiza uralensis* Fisch., and *B. macrostachya* Wall. ex Benth. were used in *jiuqu* with both high frequency and strong intersection.

### Jiuqu production process used by the Chuanqing people

The Chuanqing people have a unique production process for *jiuqu*. Jiuqu, also locally called *jiuyao*, is made using the fruits of gramineous grains, such as *Avena sativa* L. and *Eleusine coracana* (L.) Gaertn., as raw materials. Jiuyaohua (*B. macrostachya* Benth. or *Origanum vulgare* L.) and yaomu (the *jiuqu* made last year) are used as a primer, and decoction from local plants is added as excipients (among which honey should be added for FGRJ). Then, *jiuqu* is cultivated under artificial control of suitable temperature and humidity.

**Fig. 5:**
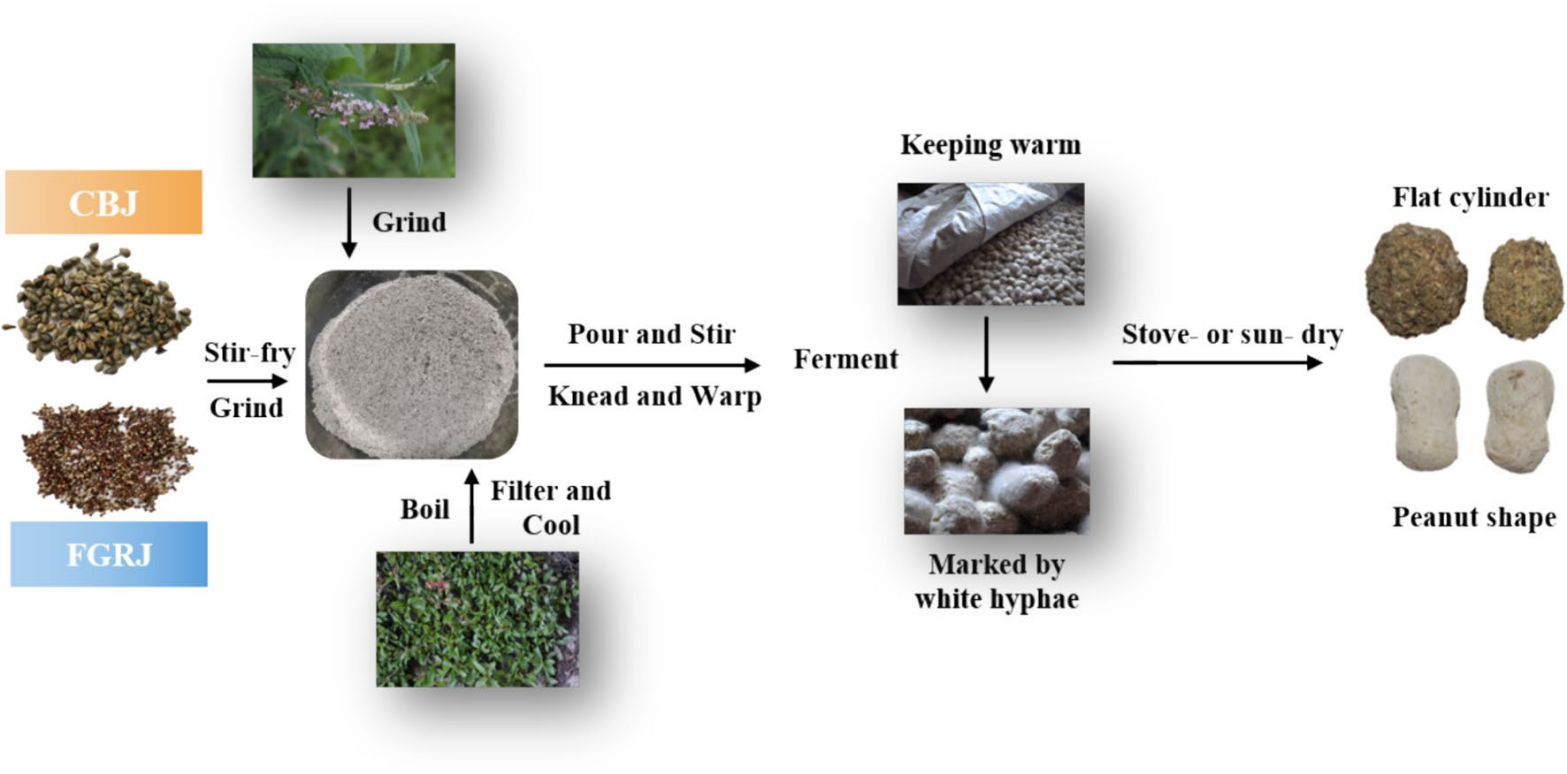
The production process for *jiuqu* performed by the Chuanqing people

The production processes of CBJ and FGRJ are different but related (Fig. 5). The steps for making *jiuqu* are as follows:

- **Step 1.** Stir-fry the substance and the raw materials (*Fagopyrum tataricum* (L.) Gaertn. for CBJ; *Avena sativa* L. or *Eleusine coracana* (L.) Gaertn. for FGRJ) until they lose moisture. Remove and cool.
- **Step 2.** Grind the fried substance and raw materials together with *B. macrostachya* Benth. or *Origanum vulgare* L.
- **Step 3.** Boil the fresh JPs with water. Pour the liquid out and cool.
- **Step 4.** Pour the cooled liquid into the powder from **Step 2**. Stir and knead into a particular shape. Then wrap with much yaomu.
- **Step 5.** After placing the *jiuqu* tightly on a dustpan covered with oat grass (or replaced by an electric blanket), cover with another layer of oat grass.
- **Step 6.** Wait for *jiuqu* to grow white hyphae, which signals successful production.
- **Step 7.** Dry the *jiuqu* in the sun and then store.

In general, three main materials are included in the production of the Chuanqing people’s jiuqu. The first is the raw material—the fruit of *Fagopyrum tataricum* (L.) Gaertn., *Avena sativa* L., or *Eleusine coracana* (L.) Gaertn. The second are the herbs— jiuyaohua (*B. macrostachya* Benth. or *Origanum vulgare* L.) and yaomu. Lastly, the excipients—compound decoction and honey.

### Unique plant ingredients and shape of jiuqu

**Fig. 6:**
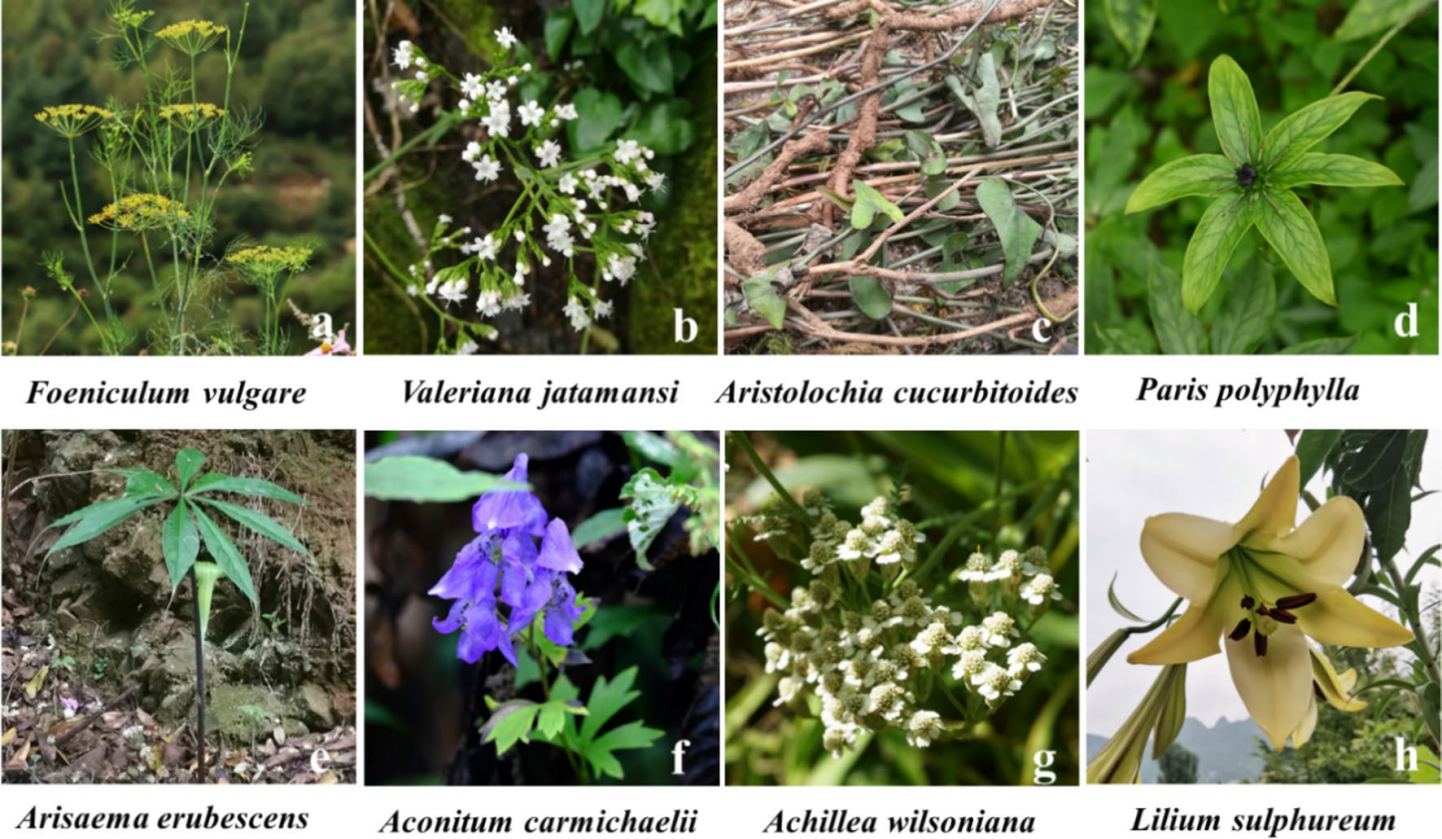
Some *Wuxiang* and *Wudu* plants in the survey area. **a**–**c** The original Wuxiang plants; **d**–**h** the original Wudu plants

During the investigation, some informants pointed out that *Wuxiang* (five plants with aromatic smells) and *Wudu* (five poisonous plants) were used in the production of *jiuqu* (Fig. 6). *Wuxiang* plants: *Foeniculum vulgare* Mill., *V. jatamansi* Jones, *Aristolochia cucurbitoides* C. F. Liang, and two other plants (no original plants were collected). Regarding the original Wuxiang plants, there are differences between different regions. For example, *Foeniculum vulgare* Mill. was replaced by *Pogostemon cablin* (Blanco) Benth. or *Syzygium aromaticum* (L.) Merr. & L.M.Perry. *Wudu* plants: *Paris polyphylla* Sm., *Arisaema erubescens* (Wall.) Schott, *Aconitum carmichaelii* Debeaux, *Achillea wilsoniana* (Heimerl) Hand.-Mazz., and *Lilium brownii* F. E. Brown ex Miellez (or *L. sulphureum* Baker ex Hook.f.). Wudu plants are often used in the production of CBJ but are rarely used or used in small doses for FGRJ production.

In addition to unique plants, the *jiuqu* shape is also exceptional. For making CBJ, *jiuqu* has a variety of shapes, from flat cylindrical to spherical. However, for FGRJ, they are peanut shaped in all regions (Fig. 5).

### Analysis of microbial diversity in jiuqu samples

**Fig. 7:**
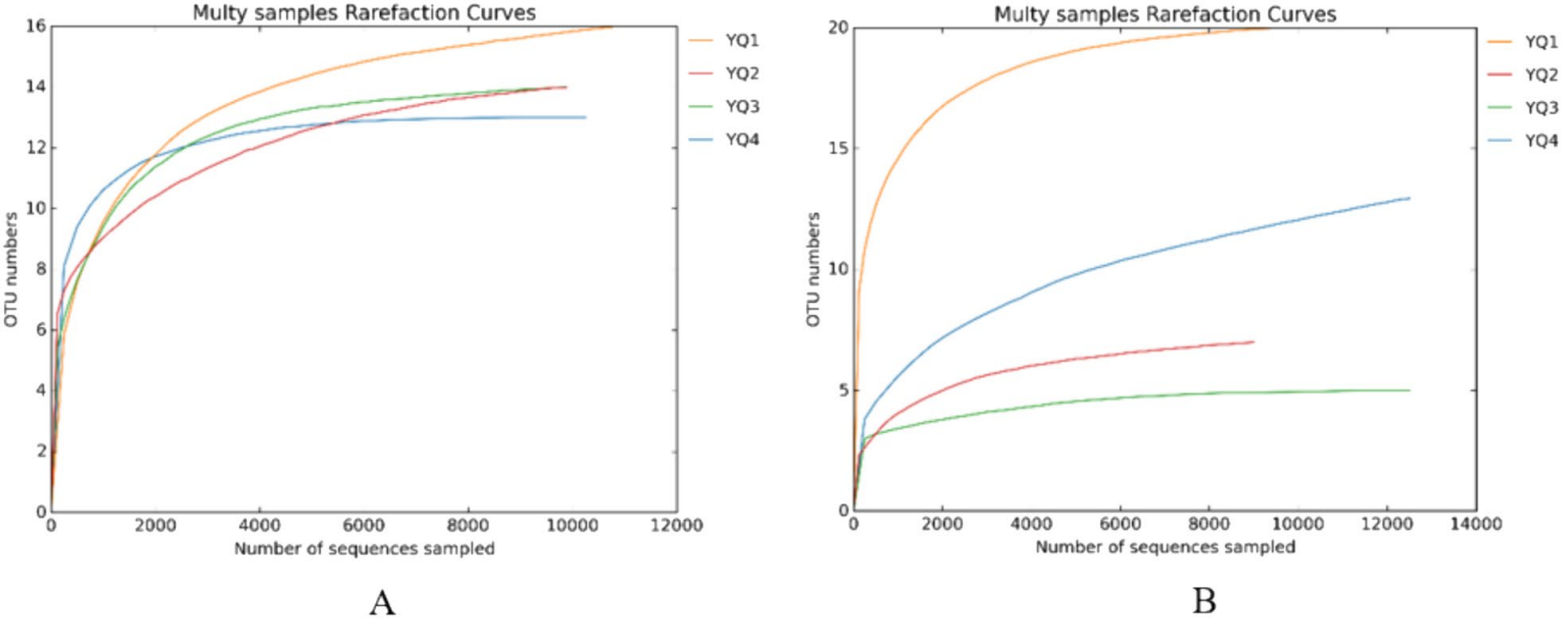
The rarefaction curves of four samples

Based on ITS1 and 16S rRNA sequencing, the numbers of effective sequences in the four samples ranged from 10,786 to 11,333 and from 9948 to 12,767 for bacteria and fungi, respectively. The OTU numbers for bacteria and fungi in each sample are shown in Table 4. The rarefaction curves were constructed with sequence and species numbers to verify whether the sequencing data were sufficient to reflect the species diversity in the four samples (Fig. 7). This suggested that the sequencing depth was adequate to represent the microbial structure and diversity of the samples. The determination of α-diversity was conducted using Chao1 and Shannon indices and coverage values (Table 4). The Chao1 indices measured the richness of species, while the Shannon indices represented species diversity. The coverage value reflects the probability of detected sequences. Table 4 shows that the Chao1 index in sample YQ1 reached 17.0000 and 20.0000 for the bacteria and fungi communities, respectively, indicating the highest species abundance. The Shannon index for samples YQ3 and YQ1 has reached 1.7123 and 2.1812 for the bacteria and fungi communities, respectively, indicating the highest species evenness.

**Table 4:** OTUs and α-diversity indices of *jiuqu* samples

ID	Bacteria				Fungi			
	OTUs	Chao1	Shannon	Coverage	OTUs	Chao1	Shannon	Coverage
YQ1	16	17.0000	1.2903	0.9998	20	20.0000	2.1812	0.9999
YQ2	14	14.0000	1.6719	0.9999	7	7.0000	0.9025	0.9999
YQ3	14	14.0000	1.7123	0.9999	5	5.0000	0.9362	1.0000
YQ4	13	13.0000	1.2964	1.0000	13	16.0000	0.9948	0.9997

The sequence coverage of the four samples was beyond 99%, indicating that more species were detected and the sequencing results could truly reflect the species abundance and diversity of the sample.

### Diversity profiles of *bacteria* and fungi communities

**Fig. 8:**
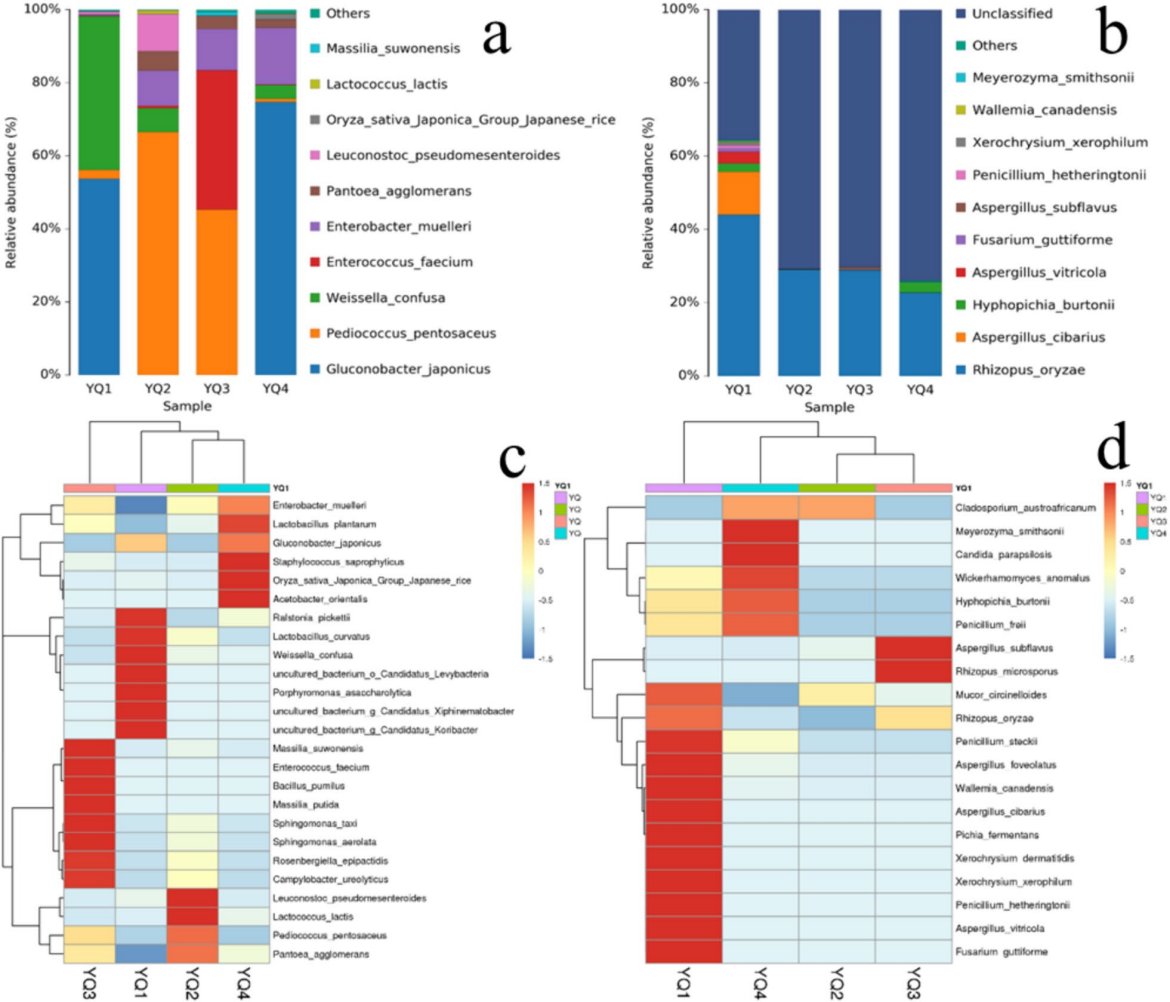
**a** Bacteria microbial community structure; **b** fungi microbial community structure; **c** cluster heatmap of bacteria species based on the microbial community profiles; **d** cluster heatmap of fungi species based on the microbial community profiles

Dissimilar bacterial and fungi communities were found at the species level for the four types of samples (Fig. 8). *Gluconobacter japonicus* was the dominant bacterial community in YQ1 and YQ4, accounting for 53.7% and 74.7%, respectively, followed by *Weissella confus*a and *Enterobacter muelleri* in YQ1 and YQ4, which accounted for 42.1% and 15.6%, respectively. *Pediococcus pentosaceus* was the dominant bacterial community in YQ2 and YQ3, accounting for 66.5% and 45.2%, respectively, followed by *Leuconostoc pseudomesenteroides* and *Enterobacter muelleri* in YQ2 and *Enterococcus faecium* and *Enterobacter muelleri* in YQ3, which accounted for 10.1%, 9.7%, 38.1%, and 11.3%, respectively.

In addition to unidentified fungal species, *Rhizopus oryzae* was also the dominant fungus in YQ1, YQ2, YQ3, and YQ4, at 44.0%, 29.0%, 29.0%, and 22.0%, respectively. *Hyphopichia burtonii* was only identified in YQ1 and YQ4, accounting for 2.3% and 3.0%, respectively. Moreover, *Aspergillus cibarius* and *Aspergillus vitricola* were found at 11.7% and 3.0% in YQ1, respectively.

Figure 8 shows the heatmap of clustering for species abundance based on the microbial community profiles. In general, many differences were found in relative species abundance among the four *jiuqu* samples. The species varied among the samples. For bacterial communities, YQ4 had a high relative abundance of *Lactobacillus plantarum*, *Staphylococcus saprophyticus*, *Acetobacter orientalis*, *Gluconobacter japonicus*, and *Enterobacter muelleri*.; YQ2 had a high relative abundance of *Pedicoccus pentosaceus*, *L. lactis*, and *Pantoea agglomerans*; YQ1 had a high relative abundance of *Ralstonia pickettii*, *L. curvatus*, *Weissella confusa, Porphyrommonas asaccharolytica*, uncultured bacterium* g Candidatus Xiphinematobacter*, and uncultured bacterium *g Candidatus Koribacter*; and YQ3 had a high relative abundance of *Massilia suwonenis*, *Enterococcus faecium*, *Bacillus pumilus*, *Massilia putida*, *Sphingomonas aerolata****,**** Rosenbergiella epipactidis*, and *Campylobacter ureolyticus*. For fungal communities, YQ3 had a high relative abundance of *Aspergillus subflavus* and *Rhizopus microspores;* YQ2 had a high relative abundance of *Cladosporium austroafricanum*; YQ4 had a high relative abundance of *Meyerozyma smithsonii*, *Candida parapsilosis*, *Wickerhamomyces anomalus*, *H. burtonii*, and *Penicillium freii*; and YQ1 had a high relative abundance of *Fusarium guttiforme*, *Aspergillus vitricola*, *Penicillium hetheringtonii*, *Xerochrysium dermatitidis*, *Pichia fermentans*, *Aspergillus cibarius*, *Wallemia canadensis*, *Aspergillus foveolatus*, *Penicillium steckii*, *Mucor circinelloides*, and *Rhizopus oryzae.*

## Discussion

### Unique jiuqu knowledge and experience of the Chuanqing people

The Chuanqing people have unique traditional fermentation knowledge and experience with *jiuqu*. To make *jiuqu*, many ethnic groups use grains as substances (raw materials are used to make *jiuqu*), such as *Avena sativa* L., *Oryza sativa* subsp*. japonica* Kato, and *Triticum aestivum* L. [14, 71]. However, in contrast with other nationalities, the Chuanqing people only use *Eleusine coracana* (L.) Gaertn. to make *jiuqu*. This usage has not been reported in domestic and foreign research; only the use of *Eleusine coracana* (L.) Gaertn. to brew alcoholic drinks has been reported [65]. Currently, the National Academy of Sciences recognizes *Eleusine coracana* (L.) Gaertn. as a potential “super grain,” and it has been found to be the most nutritional of all grains, as it is rich in protein content, fatty acids, dietary fiber, and calcium and potassium [5, 8, 26, 27, 44, 53]. In addition, it is rich in vitamin B complexes, such as thiamine, riboflavin, folic acid, and niacin [41, 43, 49]. In addition to its rich nutritional value, it also has medicinal effects. The Chinese classic medicinal *BenCaoGangMu (Compendium of Materia Medica)* once recorded that it can be used to treat indigestion [29].

The application of pungent and poisonous plants is also found to be a feature of the Chuanqing people’s jiuqu. For example, small doses of Wudu plants and *Capsicum annuum* L. are added to make CBJ. According to the informants, these plants can enhance liquor’s strong and acrid flavor. *Capsicum annuum* L. contains auxins, which are required for the growth and reproduction of various microorganisms [19]. *Paris polyphylla* Sm., *Arisaema erubescens* (Wall.), *Aconitum carmichaelii* Debeaux, *Achillea wilsoniana* (Heimerl) Hand. -Mazz., and *L. brownii* F. E. Brown ex Miellezan (or *L. sulphureum* Baker ex Hook. f.) all contain toxicity [3, 35, 45, 48, 63, 67, 68]. The informants believe that the application of Wudu plants can inhibit the growth of miscellaneous bacteria during the fermentation process. Poisonous plants are also consumed in other parts of China, for example, most people in certain villages in the Taibai Mountains of the Qinling range (Shaanxi) regularly consume *Aconitum* to keep body warm every winter, as well as to get the same nutrients as other staples 24.

In addition, to make FGRJ, the Chuanqing people also knead the *jiuqu* into a peanut shape, which is very different from other spherical or cylindrical shapes of *jiuqu*. We speculate that this may be due to the following reasons. First, the peanut shape of FGRJ can be easily distinguished from other *jiuqu*. Second, due to the high market competitiveness of *jiuqu*, the unique peanut shape can give consumers a different impression, thereby promoting sales and trademarks.

### Application of Jiuqu in the medicinal health care, family livelihood, and cultural customs of the Chuanqing people

Through investigation and research, the Chuanqing people’s *jiuqu* has become popular in local health care, family livelihood, and cultural customs. Not only can the Chuanqing people’s *jiuqu* be brewed for Chinese *jiuqu* and fermented glutinous rice, but it is also featured strongly in the health care of the local people. Indigestion diseases are common and frequent occurrences in the local clinics of the Chuanqing people [37]. The prevalence of indigestion in Asia is as high as 8–30% [13]. As a traditional medicine, convenient and cheap *jiuqu* can significantly meet the needs of the local people for treating indigestion diseases. Moreover, fermented glutinous rice is closely related to the Chuanqing people’s daily lives. For example, when the local farmers work outdoors, they will drink fermented glutinous rice to supply nutrition and water.

Fei Xiaotong believed that rural handicrafts and agricultural production were closely integrated into the traditional peasant society to support farming families’ livelihoods [7]. In the Chuanqing people’s daily lives, *jiuqu* ensures the local people’s material life as a cottage industry. In the 1970s and 1980s, many local people made and sold *jiuqu* to support their families. In recent years, the price of *jiuqu* has been usually about 1–2 yuan (US$0.16 ~ 0.32) for every piece, and the annual profit has been about 2000–3000 yuan (US$290 ~ 435). According to the Statistical Bulletin of Nayong County’s National Economic and Social Development in 2021, compiled by the Nayong Survey Office of the National Bureau of Statistics, the per capita disposable income of rural households in Nayong County was 11,829 yuan (US$1,715.20) (https://www.gznayong.gov.cn). The income from *jiuqu* can be seen to play a vital role in local families and in greatly improving the local people’s income and living standards.

Moreover, traditional *jiuqu* is an integral part of the Chuanqing people’s culture. Chinese *baijiu* and fermented glutinous rice are also indispensable substances in the local people’s lives. The Chuanqing people’s Chinese *baijiu* and fermented glutinous rice have a variety of social and cultural functions, such as carrying and transmitting history and culture and transmitting emotion and information. The Chuanqing people use fermented glutinous rice to entertain guests from afar, to express blessings at weddings, and to celebrate traditional festivals, and fermented glutinous rice is spilled onto the ground as a ritual to pay tribute to deceased loved ones at funerals and during the Ching Ming Festival. In short, drinking is an expression of Chuanqing people’s culture and customs.

### Microbial community diversity analysis of FGRJ

The present study is the first to explore the microbial mystery behind FGRJ. In this study, FGRJ was sequenced for the first time using the PacBio platform to understand the microbial community.

The most prevalent bacteria belonging to FGRJ were *Gluconobacter japonicu, Pediococcus pentosaceus, Enterococcus faecium,* and *Weissella confusa*. *Gluconobacter japonicus* was the most predominant bacterium in homemade YQ1 and YQ4, while *Weissella confusa* accounted for a large fraction in YQ1. *Pediococcus pentosaceus* was the most predominant bacterium in purchased YQ2 and YQ3, while *Enterococcus faecium* accounted for a large fraction in YQ3. A variety of factors, such as substance, herbs, and microhabitat contained in niches, might have contributed to these differences [57]. *Pediococcus pentosaceus* and *Enterococcus faecium* confusa were present in fermented foods and have been recognized as probiotic species (Jiang et al., 2021; Kim et al., 2016). Probiotics have been demonstrated to promote the treatment of disease and to improve body balance.

*Rhizopus oryzae* was the main fungus found in the four samples. It could release various enzymes to accelerate starch saccharification and enhance the utilization of starch. Moreover, it could inhibit bacterial growth and improve the flavor of fermented glutinous rice [33]. Most of the fungi found were unclassified. This may be because these fungi have never been given a name or the database of this platform is not rich enough to include this part of the fungus.

Some studies have argued that these plants, as traditional herbs and microbes, can establish synergistic relationships. Traditional herbs can improve microorganisms’ nutrition, inhibit miscellaneous bacteria and antioxidation, promote the growth of yeast and mold, and change the aroma of alcoholic drinks [59, 64]. Simultaneously, proteases, cellulases, and other enzymes produced by microbial metabolism can destroy the cellular structure of herbs, promoting the dissolution of the active ingredients. Subsequently, these herbs can be degraded into small molecules, such as sugars and amino acids, by microorganisms and thus greatly improve their efficacy [17]. At the same time, the microorganisms can also decompose toxic substances and reduce side effects in traditional Chinese medicine [31]. Therefore, further studies are required to determine how these plants affect *jiuqu*’s quality, medicinal efficacy, and brewing.

The history of adding herbs to *jiuqu* goes back a long way in China, but little research has been conducted on the interactions between folk herbs and microbes; thus, further studies are needed.

### Predicament facing the Chuanqing people’s knowledge of traditional CBJ

The data were analyzed by SPSS and show that CBJ knowledge has mainly been mastered by elderly people. However, with the deepening of social aging and the continuous decline in the number of rural labor force resources, the transmission of CBJ knowledge through history faces a serious crisis. The spread of conservativeness and the limited and broken inheritors are the main factors driving this crisis. First, most holders of CBJ knowledge are illiterate farmers. Oral practice is a common way for them to acquire knowledge. Thus, once knowledge holders’ memories become clouded or die, this information can be lost. Next, the Chuanqing people live in isolated mountain areas, where transportation is inconvenient, and CBJ production is a dominant means of livelihood. Thus, they tend to be conservative in teaching living skills. Finally, the traditions and livelihoods of Chuanqing communities are being shaped by rapid globalization, with young people moving to the big city to earn a living.

Knowledge of the Chuanqing people’s *jiuqu* belongs to not only individual, but also collective and multi-species, spanning generations and connecting the present and the past 42. The 15th meeting of the Conference of the Parties to the Convention on Biology Diversity (COP15) noted that protecting traditional knowledge would provide an opportunity for future cultural diversity conservation and sustainable development of indigenous communities around the world. Therefore, to implement these provisions of the convention, it is imperative to conserve the traditional knowledge and craftsmanship of CBJ as an important practice. On the one hand, taking advantage of the new media wave, *jiuqu* makers are using online social media platforms to post short videos, not only to record knowledge about *jiuqu* but also to increase additional income opportunities. Establishment of a multimedia database, such as audio, video, images, and data, can be used to stockpile CBJ knowledge. This would remove spatial barriers and time constraints, thus protecting this information forever. On the other hand, the government provides financial and policy support to the CBJ talents and formulates patent and secret trade protection. Meanwhile, they also cultivate inheritors and constantly improve and innovate CBJ production theory and practice. In conclusion, with the help of an established database establishment and government assistance, CBJ knowledge could be well protected.

## Conclusion

This study is the first ethnobotanical survey to record traditional JPs and *jiuqu*-making techniques among the Chuanqing people of northwestern Guizhou, China. A total of 57 species belonging to 51 genera and 32 families were found to be used to make CBJ and FGRJ in the Chuanqing people communities. Jiuqu has dual medicinal and brewing functions. This study is the first to document *E. coracana* (L.) Gaertn. as a substance for making *jiuqu*. Wuxiang and Wudu plants are used in *jiuqu* making to improve the growth and reproduction of microorganisms. The main bacteria are *Gluconobacter japonicus* (YQ1, YQ4) and *Pediococcus pentosaceus* (YQ2, YQ3), and the main fungus is *Rhizopus oryzae*. Additional studies are required to explore the relationship between microorganisms and the medicinal value of *jiuqu*. Moreover, *jiuqu* plays an essential role in the households and cultural practices in the Chuanqing people’s communities. Nevertheless, with the increasing age of *jiuqu* knowledge holders, CBJ knowledge is facing a severe inheritance crisis. Thus, this study proposes static and dynamic conservation modes to prevent the disappearance of traditional *jiuqu* knowledge through the establishment of a database as well as governmental peer-to-peer assistance. This research contributes to the protection and inheritance of traditional *jiuqu* knowledge of the Chuanqing people. It lays the foundation for further research on the microbiology, nutrition, and metabolomics of the Chuanqing people’s *jiuqu*.

## Data Availability

The datasets used and/or analyzed during the current study are available from the corresponding author on reasonable request.

## Abbreviations

CBJ: Chinese *Baijiu Jiuqu*
FGRJ: Fermented glutinous rice *Jiuqu*
JPs: *Jiuqu* Plants
OTUs: Operational taxonomic units
